# Personality Type, Emotional Intelligence, and Successful Matching Into Emergency Medicine Residency Programs

**DOI:** 10.7759/cureus.113751

**Published:** 2026-07-31

**Authors:** Areej N Al Balkhi, Ayman M AlGhazzi, Ahmad M AlSaif, Toleen F Shaikh, Kholoud A Babkair, Thamir o AlSayed, Mohammad A Zaher

**Affiliations:** 1 Research, King Fahad Medical City, Riyadh, SAU; 2 Emergency Medicine, Prince Mohammed Bin Abdulaziz Hospital, Riyadh, SAU; 3 Emergency Medicine, King Fahad Specialist Hospital, Dammam, SAU; 4 Emergency Medicine, King Abdulaziz Medical City, Ministry of National Guard Health Affairs, Jeddah, SAU; 5 Emergency Medicine, King Fahad Hospital of the University, Alkhobar, SAU

**Keywords:** academic performance, emergency medicine residency, emotional intelligence, personality traits, residency match, saudi arabia

## Abstract

Background

Personality traits and emotional intelligence (EI) have been proposed as important non-cognitive predictors of performance and well-being in medical training. However, their role in residency selection remains unclear. This study examined the relationship between personality, EI, and successful matching into Saudi emergency medicine (EM) residency programs.

Methods

A prospective study was conducted involving all applicants to the 2019 Saudi Board of Emergency Medicine (SBEM) phase 2 residency match. Data collected included demographics, composite academic scores (grade point average (GPA), Saudi Medical Licensing Examination (SMLE), and portfolio scores), EI measured using the Trait Emotional Intelligence Questionnaire-Short Form (TEIQue-SF), and personality traits assessed using the NEO Five-Factor Inventory (NEO-FFI-3). Logistic regression models were used to analyze associations with residency acceptance.

Results

A total of 151 applicants (mean age: 25.6 ± 1.9 years; 57.0% male) were included in the analysis, of whom 125 (82.8%) successfully matched into an EM residency. Univariate analysis showed that younger age, being unmarried, higher composite scores (primarily driven by GPA), and higher TEIQue well-being and emotionality sub-scores were associated with successful matching. In multivariate analysis, only composite score (OR = 1.25, 95% CI: 1.00-1.56, p = 0.046) and GPA (OR = 1.13, 95% CI: 1.02-1.26, p = 0.017) remained significant predictors. Personality traits and overall EI did not show independent associations with residency acceptance.

Conclusion

Academic performance, particularly GPA, remains the strongest predictor of successful matching into Saudi EM residency programs. Although personality traits and EI were not independently associated with acceptance, well-being may play a supportive role. These findings underscore the continued emphasis on cognitive metrics in residency selection, not as an argument against assessing non-cognitive attributes, but as evidence of a gap between the value program directors (PDs) place on personality and interview impression and the lack of structured tools currently used to measure them, pointing to a clear opportunity to develop standardized non-cognitive assessments for EM applicant selection.

## Introduction

Understanding the factors that contribute to the performance of physicians-in-training has long been a central focus of medical education research [1]. Two major, complementary components are widely theorized to influence this performance: cognitive abilities and non-cognitive attributes (e.g., personality traits, emotional intelligence (EI), communication skills, professionalism, resilience, and interpersonal competence). Cognitive abilities, which are often reflected by examination scores, have traditionally been the primary metric used to evaluate medical students and physicians in both undergraduate and postgraduate settings, including residency selection [2,3]. In contrast, while non-cognitive attributes are increasingly recognized as vital to success and well-being in medical training, there has been relatively limited effort to adopt standardized methods for their assessment [1].

Among non-cognitive predictors, personality traits have received considerable attention in the context of medical education and clinical practice. Several studies suggest that personality assessments can offer valuable insights during medical school admissions and should be more systematically considered throughout physician training [2,4]. For example, while intellectual abilities alone may explain approximately 35% of the variance in medical school performance, adding personality measures can increase the explained variance to as much as 75% [2]. Furthermore, personality traits have been linked to differences in clinical performance, board examination scores among internal medicine residents [5], and competency levels in surgical and anesthesiology programs [6,7]. Personality also impacts physician well-being; self-identification with certain personality types has been associated with better perceived professional identity, lower emotional exhaustion, and improved outcomes in anxiety, depression, job satisfaction, and career commitment [8].

Similarly, EI, which is defined as the ability to perceive, understand, and regulate one’s own emotions as well as those of others [9], has emerged as another important non-cognitive factor influencing various aspects of medical education. Multiple studies have explored the relationship between EI and outcomes such as medical school admissions, academic performance, physician burnout, job satisfaction, and even patient satisfaction [10-13]. While findings generally suggest a modest positive relationship between EI and academic performance [10,11], EI training has also been shown to enhance patient satisfaction in certain specialties, such as otolaryngology [12].

However, the role of EI is not universally beneficial. Recent literature has indicated that individuals with high EI may experience higher levels of acute stress and slower cortisol recovery in socially demanding situations [13]. Chronic stress in emotionally intelligent individuals may also lead to internalizing behaviors [14]. Moreover, some studies have examined the intersection between EI and maladaptive traits such as psychopathy and narcissism. While some evidence suggests EI could be used manipulatively in individuals with psychopathic tendencies, other findings point to a significant negative correlation between EI and psychopathy [15,16].

Despite the growing body of evidence supporting the predictive value of personality and EI, some medical educators remain skeptical about their utility in structured assessments. Many continue to rely on traditional, unstructured tools such as face-to-face interviews, reference letters, and personal statements. While these methods aim to incorporate a “human touch” into the selection process, they are often limited by subjectivity and lack of standardization [1].

In the field of emergency medicine (EM), the importance of accurately assessing personality traits and EI may be even more pronounced. The emergency department is a dynamic, fast-paced, and high-stakes environment that requires strong interpersonal skills, emotional regulation, and effective communication with a wide range of stakeholders, including patients, families, nurses, emergency medical services (EMS) personnel, administrators, and physicians from other specialties [3]. Given the stakeholders, including the consistent identification of non-cognitive attributes in EM applicants, may be crucial not only for predicting job performance but also for evaluating a candidate’s capacity to thrive in this complex setting. Nevertheless, limited evidence exists regarding the impact of personality traits and EI on successful matriculation into EM residency programs.

This study aims to evaluate the relationship between two key non-cognitive attributes, personality type and EI, and successful matching into EM residency programs in Saudi Arabia, specifically among applicants to phase 2 of the Saudi Board of Emergency Medicine (SBEM) match.

## Materials and methods

Study design and participants

We conducted a prospective study that included all SBEM applicants for the 2019 phase 2 residency program match in all regions of the Kingdom. The Saudi Arabian residency match is composed of two phases. In phase 1, applicants compete for specialty choices based on a composite score (CS) set by the Saudi Commission for Health Specialties (SCFHS). The CS is comprised of three components: the medical school global point average (GPA), which contributes 50% of the CS; the Saudi Medical Licensing Examination (SMLE) score, which contributes 30% of the CS; and the portfolio score, which is a point system created by the SCFHS based on scholarly and extracurricular achievements and contributes 20% of the CS. In phase 2 of the match, candidates nominated for specialty training seats in phase 1 are interviewed by residency training centers of that specialty. Rank lists from both the applicants and the centers ultimately determine which center (if any) the applicant matches with. Phase 2 applicants in this study were Saudi medical school graduates who, having been allocated an EM training seat in phase 1 based on their CS, were subsequently interviewed by EM residency training centers. The study therefore included only applicants competing specifically for EM residency positions and excluded candidates applying for other specialties. "Acceptance" (successful matching) is defined throughout this study as being formally assigned to an EM residency training seat following phase 2 interviews and rank-list processing, rather than merely being interviewed or ranked without an eventual placement.

Materials

The data collected for this study fell into four domains: demographic data; academic performance data, including the SCFHS CS, its components, and the candidates’ phase 2 residency program match results; EI scores measured through the Trait Emotional Intelligence Questionnaire-Short Form (TEIQue-SF); and personality trait scores measured through the NEO Five Factor Inventory (NEO-FFI-3) [17,18]. The portfolio score component of the CS reflects documented scholarly and extracurricular activities, including research publications, completed courses and workshops, and volunteer or leadership activities, which are reviewed and scored by SCFHS-appointed referees. Additionally, one training center in Riyadh provided candidate interview score data that were included in a post hoc subgroup analysis. The interview score included points for perceived communication skills, professionalism, fitness for the program, and response to a clinical scenario.

NEO-FFI-3

The NEO-FFI-3 is a 60-item questionnaire that measures five personality domains, as originally described in the NEO-FFI manual [17]: openness (fantasy, esthetics, feelings, ideas, actions, imagination, preference for variety, curiosity, and intellectual qualities), conscientiousness (competence, dutifulness, self-discipline, deliberation, and order), extraversion (sociability, warmth, activity, positive emotions, and assertiveness), agreeableness (trust, compliance, straightforwardness, altruism, and modesty), and neuroticism (anxiety, anger, depression, impulsiveness, and vulnerability). It has been validated in medical education settings [1].

TEIQue-SF

The TEIQue-SF is a 30-item, shortened version of the original Trait Emotional Intelligence Questionnaire developed by Petrides [18], measuring the following facets of trait EI: well-being (self-esteem, trait happiness, and trait optimism), self-control (emotion regulation, stress management, and low impulsivity), emotionality (emotion perception, trait empathy, emotion expression, and relationships), and sociability (assertiveness, emotion management, and social awareness). Both versions of the TEIQue have been extensively validated [19].

Procedures

Demographic data, along with the study instruments (TEIQue-SF and NEO-FFI-3), were collected through an email campaign survey using the Alchemer online survey platform (Alchemer LLC, Louisville, CO). Participants received an email invitation to complete the survey, and academic performance data were obtained from the regional program directors (PDs).

Statistical analysis

All study variables were included in univariate binary logistic regression models and were then combined into a multivariable model if they were found to be significant (p < 0.05). A post hoc subgroup analysis was conducted utilizing interview scores provided by a single residency training center in Riyadh. A Bonferroni correction was applied to the CS and its components (GPA, SMLE, and portfolio) to account for multiple comparisons among related academic variables (adjusted α = 0.025 for the CS; adjusted α = 0.017 for GPA, SMLE, and portfolio individually). For all other variables, p < 0.05 was considered statistically significant. Statistical analyses were performed using IBM SPSS Statistics version 27 (IBM Corp., Armonk, NY).

## Results

**Figure 1:**
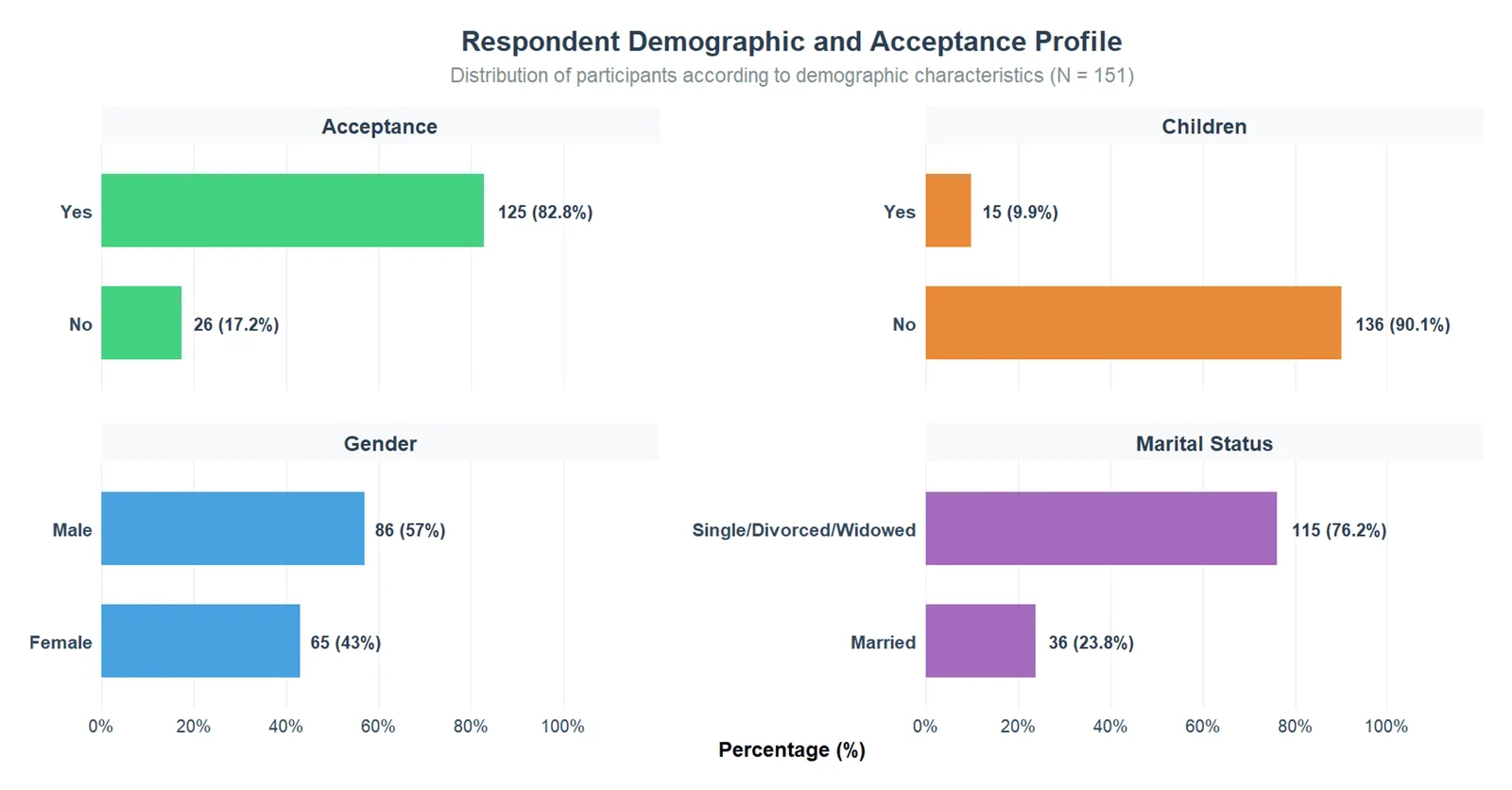
Distribution of participants by demographic characteristics (N = 151)

A total of 151 participants were included in the study, with ages between 24 and 34 years (mean ± SD, 25.6 ± 1.9 years). Of these, 57% were males, and 82.8% were eventually enrolled in the Saudi EM training program (Table 1). Moreover, the demographic distribution of participants is presented in Figure 1.

**Table 1:** Demographic and descriptive data of the participants (N = 151) Continuous variables are presented as mean ± standard deviation (SD), whereas categorical variables are presented as frequency (n) and percentage (%). GPA: Grade point average; SMLE: Saudi Medical Licensing Examination; SD: Standard deviation.

Characteristics	Category	Mean ± SD / n (%)
Age (years)	—	25.6 ± 1.9
Composite score	—	75.49 ± 2.60
GPA score	—	85.67 ± 5.30
SMLE score	—	75.04 ± 4.40
Portfolio score	—	12.27 ± 1.70
Acceptance	Yes	125 (82.8)
	No	26 (17.2)
Gender	Female	65 (43.0)
	Male	86 (57.0)
Marital status	Single/divorced/widowed	115 (76.2)
	Married	36 (23.8)
Children	Yes	15 (9.9)
	No	136 (90.1)

EI characteristics using TEIQue-SF

The scores of those who participated in the survey ranged between 2.27 and 6.8 IQ points for the global trait, whereas the score ranges of the factors are shown in Table 2. It is noteworthy to mention that the candidates scored relatively higher on the well-being scale than all the other factors. However, their responses on this particular factor had the highest variability. The 95% confidence intervals (CIs) indicate that the difference between well-being and the other TEIQue-SF sub-scales was significant, as their intervals did not overlap (Table 2).

**Table 2:** Descriptive analysis of the TEIQue-SF (N = 151) TEIQue-SF: Trait Emotional Intelligence Questionnaire-Short Form; SD: Standard deviation; CI: Confidence interval.

TEIQue-SF Domain	Minimum	Maximum	Mean ± SD	95% CI of the Mean
Well-being	1.00	7.00	5.58 ± 1.04	5.41–5.75
Self-control	2.83	6.83	4.80 ± 0.87	4.66–4.94
Emotionality	1.63	6.88	5.03 ± 0.90	4.89–5.17
Sociability	2.50	6.83	5.13 ± 0.85	4.99–5.27
Overall TEIQue-SF score	2.27	6.80	5.18 ± 0.69	5.07–5.29

Personality characteristics using NEO-FFI-3

Most candidates fell into the average category for neuroticism, average-to-high on extraversion, openness, and conscientiousness, and low-to-average on agreeableness. The 95% CIs indicate that the agreeableness score range was the only one significantly lower than the others (Table 3).

**Table 3:** Descriptive analysis of the NEO Five-Factor Inventory (NEO-FFI-3) CI: Confidence interval.

Personality Domain	Category	n (%)	Minimum	Maximum	Mean	95% CI of the Mean
Neuroticism	Very low	2 (1.3)	25	70	51.62	50.13–52.73
	Low	24 (15.9)				
	Average	84 (55.6)				
	High	32 (21.2)				
	Very high	9 (6.0)				
Extraversion	Very low	0 (0.0)	37	75	57.59	56.12–58.83
	Low	7 (4.6)				
	Average	52 (34.4)				
	High	63 (41.7)				
	Very high	29 (19.2)				
Openness	Very low	2 (1.3)	32	75	56.04	54.67–57.42
	Low	10 (6.6)				
	Average	58 (38.4)				
	High	63 (41.7)				
	Very high	18 (11.9)				
Agreeableness	Very low	10 (6.6)	25	65	46.50	44.99–47.85
	Low	54 (35.8)				
	Average	63 (41.7)				
	High	24 (15.9)				
	Very high	0 (0.0)				
Conscientiousness	Very low	5 (3.3)	25	75	52.35	50.87–53.87
	Low	23 (15.2)				
	Average	64 (42.4)				
	High	48 (31.8)				
	Very high	11 (7.3)				

Associations between the variables

All study variables were first included in univariate binary logistic regression models, then combined into a multivariable model if they came back significant (p < 0.05). Following that approach, variables that came back significant from the univariate analysis were age and being married, as they are negatively associated with being accepted (p = 0.02 and p = 0.02, respectively). The CS (GPA, SMLE, and portfolio score) was positively associated with acceptance (p = 0.015) and was found to be driven by the GPA score (p = 0.02). The TEIQue-SF well-being and emotionality sub-scores were also positively associated with acceptance (p = 0.04 and p = 0.39, respectively). The associations between all the predictor variables and the outcome (acceptance) on the univariate analysis are shown in Table 4.

**Table 4:** Univariate analysis model of all variables P < 0.05 was considered statistically significant. ^†^ Reference categories: Female (gender), Married (marital status), and No (children). ^‡^ Bonferroni-adjusted significance level: α = 0.025. ^§^ Bonferroni-adjusted significance level: α = 0.017. B: Regression coefficient; SE: Standard error; OR: Odds ratio; CI: Confidence interval.

Variables	B	SE	Wald	df	P-value	OR (Exp(B))	95% CI
Age (years)	-0.227	0.099	5.282	1	0.022	0.797	0.657–0.967
Gender (Male vs Female^†^)	-0.854	0.477	3.206	1	0.073	0.426	0.167–1.084
Marital status (Single/Divorced/Widowed vs. Married^†^)	-1.076	0.456	5.579	1	0.018	0.341	0.140–0.833
Children (Yes vs No^†^)	-0.206	0.685	0.090	1	0.764	0.814	0.213–3.116
Composite score^‡^	0.260	0.107	5.901	1	0.015	1.298	1.052–1.601
GPA^§^	0.137	0.045	9.341	1	0.002	1.147	1.050–1.253
SMLE^§^	0.072	0.048	2.249	1	0.134	1.074	0.978–1.180
Portfolio^§^	-0.159	0.124	1.642	1	0.200	0.853	0.669–1.088
Well-being	0.390	0.189	4.237	1	0.040	1.477	1.019–2.141
Self-control	0.032	0.247	0.017	1	0.897	1.032	0.636–1.676
Emotionality	0.493	0.239	4.255	1	0.039	1.637	1.025–2.614
Sociability	0.263	0.250	1.107	1	0.293	1.301	0.797–2.125
Neuroticism	-0.032	0.028	1.379	1	0.240	0.968	0.917–1.022
Extraversion	0.038	0.027	2.031	1	0.154	1.039	0.986–1.095
Openness	0.020	0.025	0.639	1	0.424	1.020	0.971–1.072
Agreeableness	0.002	0.025	0.010	1	0.920	1.002	0.955–1.052
Conscientiousness	-0.002	0.023	0.004	1	0.947	0.998	0.954–1.045

When the significant variables above were included in a multivariable regression model, only CSs (driven by GPA) maintained their significance (p = 0.046) (Table 5). Table 6 displays the output from the multivariable logistic regression model wherein the CS was substituted with the GPA score. After controlling for age, well-being, emotionality, and marital status, GPA was found to be the only significant predictor of admission to the Saudi Emergency Medicine Training Program (B = 0.126, OR = 1.134, 95% CI: 1.023-1.258, p = 0.017). This means that a one-unit increase in GPA increases the odds of acceptance by 13.4%, with all other variables held constant. On the other hand, the effect sizes of age, well-being, emotionality, and marital status were insignificant (p > 0.05). Even if participants who are married had higher odds of being accepted to the program (OR = 2.415), this relationship was statistically insignificant (p = 0.116). In general, these results imply that GPA serves as an independent predictor of acceptance, and the other variables used in this study have no significant independent relationships with acceptance.

**Table 5:** Multivariate regression model with composite scores P < 0.05 was considered statistically significant. B: Regression coefficient; OR: Odds ratio; CI: Confidence interval.

Variables	B	SE	Wald	df	P-value	OR (Exp(B))	95% CI
Age (years)	-0.073	0.118	0.376	1	0.540	0.930	0.737–1.173
Marital status (Single/Divorced/Widowed vs. Married^†^)	-0.900	0.529	2.898	1	0.089	0.407	0.144–1.146
Composite score	0.223	0.111	3.989	1	0.046	1.249	1.004–1.555
Well-being	0.178	0.243	0.536	1	0.464	1.195	0.742–1.923
Emotionality	0.465	0.316	2.162	1	0.141	1.592	0.857–2.960

**Table 6:** Multivariate regression model with composite scores replaced by GPA P < 0.05 was considered statistically significant. ^†^ Bonferroni-adjusted significance level: α = 0.017. B: Regression coefficient; OR: Odds ratio; CI: Confidence interval.

Variables	B	SE	Wald	df	P-value	OR (Exp(B))	95% CI
Age (years)	0.013	0.132	0.010	1	0.920	1.013	0.782–1.313
Well-being	0.192	0.243	0.625	1	0.429	1.211	0.753–1.949
Emotionality	0.393	0.314	1.566	1	0.211	1.482	0.800–2.745
GPA^†^	0.126	0.053	5.733	1	0.017	1.134	1.023–1.258
Married status	0.882	0.561	2.469	1	0.116	2.415	0.804–7.252

A post hoc analysis was carried out for the Riyadh subgroup, adding the interview total and category scores (communication skills, professionalism, fitness for the program, and response to a clinical scenario) as variables. Yet that analysis still yielded no significant predictors even when applying a looser alpha, as shown in the multivariate model in Table 7.

**Table 7:** Multivariate regression analysis of the Riyadh subgroup P < 0.05 was considered statistically significant. B: Regression coefficient; OR: Odds ratio; CI: Confidence interval.

Variables	B	SE	Wald	df	P-value	OR (Exp(B))	95% CI
Married status	2.155	0.874	6.073	1	0.014	8.625	1.554–47.864
Portfolio score	-0.485	0.234	4.293	1	0.038	0.616	0.389–0.974

The results from the multivariable logistic regression analysis for the Riyadh subgroup are shown in Table 7. It was observed that both marital status and portfolio score were statistically significant predictors of acceptance in the Saudi Emergency Medicine Training Program. In particular, those who reported their marital status as "married" were 8.63 times more likely to be accepted than those from the reference category (OR = 8.625, 95% CI: 1.554-47.864, p = 0.014). However, contrary to the above predictor, the portfolio score showed a negative relationship with acceptance (B = −0.485, OR = 0.616, 95% CI: 0.389-0.974, p = 0.038). In detail, for every one-unit rise in the portfolio score, there was a 38.4% decrease in the likelihood of being accepted, adjusting for the effect of the remaining variable in the equation.

**Figure 2:**
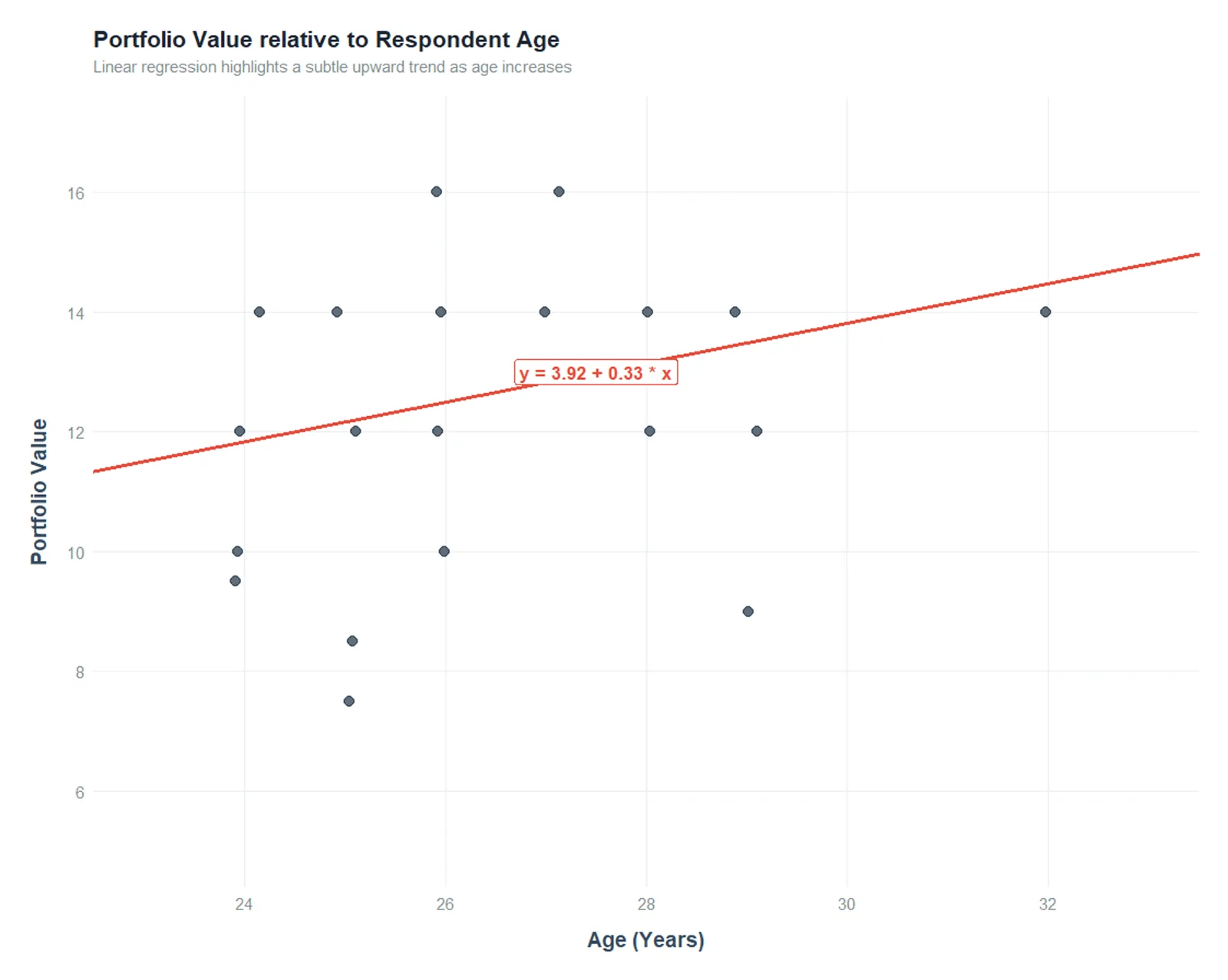
Scatter plot showing the correlation between portfolio score and age

When the acceptance rate was stratified by gender and marital status, married males had 2.5 to 5 times the rejection rate compared to the other candidates, and this difference was statistically significant for both the overall sample and the Riyadh subgroup using the chi-square test (p = 0.043 for both) (Figure 2).

Given the borderline p-value for portfolio score, we explored potential confounders. Since higher portfolio scores tend to be obtained by older applicants, age was examined as a possible confounder. A weak but statistically significant linear correlation was found between portfolio score and age (Pearson r = 0.30, p = 0.006). After controlling for age, partial correlation analysis confirmed a statistically significant confounding effect (p = 0.044).

When acceptance was stratified by a combined gender-and-marital-status variable (married female, other female, single male, or married male), married male applicants showed a substantially higher rejection rate than the other categories. This association was statistically significant in both the overall sample (Wald score χ² = 8.137, df = 3, p = 0.043) and the Riyadh subgroup (Wald score χ² = 8.170, df = 3, p = 0.043), as detailed in Table 8.

**Table 8:** Association between the combined gender-and-marital-status variable and acceptance Values represent the Wald chi-square statistic (df = 3) for the four-level gender-by-marital status categorical variable (married female, other female, single male, and married male), derived from univariate logistic regression before model entry and used to test the overall association with acceptance. P < 0.05 was considered statistically significant.

Sample	Predictor	Wald Score χ²	df	P-value
Overall sample (N = 151)	Gender × marital status (4 categories)	8.137	3	0.043
Riyadh subgroup (N = 82)	Gender × marital status (4 categories)	8.17	3	0.043

## Discussion

In our study, the CS and GPA were positively associated with acceptance into Saudi EM residency programs, with age acting as a confounding factor. Younger applicants were more likely to be accepted, while being married was negatively associated with acceptance in the overall univariate analysis. The well-being sub-score of EI demonstrated a positive association with acceptance. Based on our findings, academic performance, specifically the CS and GPA, was objectively the strongest predictor of acceptance into EM residency programs.

This academic emphasis is unlikely to be unique to EM training: GPA- and score-driven selection is a widely documented pattern across residency specialties more broadly, and factors not captured in this dataset, such as interview structure, letters of recommendation, and program-specific priorities, are also likely to shape outcomes elsewhere. It is also possible that this pattern partly reflects a broader cultural emphasis on academic achievement as a primary marker of merit within Saudi (and more broadly Gulf/Middle Eastern) educational systems, rather than a feature specific to EM selection or this cohort. Comparative studies conducted in residency systems with different structural and cultural selection norms would help clarify whether these findings generalize beyond the Saudi context.

These results contrast with a previous study in which Saudi EM PDs reported that personality traits and overall interview impression were the most critical criteria for selecting residents [20]. This discrepancy may be explained by several factors. First, PDs may not use structured or standardized tools for assessing EI and personality, instead relying on subjective interview impressions. Second, implicit bias may influence PDs’ decisions, as they might subconsciously associate high academic performance with favorable personality characteristics, inadvertently reinforcing academic metrics as selection determinants. Third, the personality traits most valued by PDs may not be adequately captured by the validated tools used in this study, despite their established reliability in medical education contexts. Fourth, the diversity of EM applicants may make it difficult for PDs to prefer a single personality or EI profile. Fifth, the relationship between EI, personality, and acceptance may differ across specialties, warranting future comparative studies. Finally, variability in PD selection practices may dilute potential associations between personality, EI, and acceptance outcomes.

The lack of a clear association between EI and acceptance may reflect a gap in current educational and training systems, which may not be structured to adequately nurture or assess EI in medical students. Relying on subjective interview impressions as a proxy for EI could lead to inaccuracies in determining candidates’ suitability for EM training. Our findings highlight the need for medical education programs to better integrate EI development and structured assessment into undergraduate and postgraduate curricula.

Previous research has emphasized the importance of EI in managing stress and promoting patient-centered care, both of which are essential competencies in EM [21,22]. However, Papanagnou et al. found no correlation between EI and residents’ training status [23]; the study also found that EI scores decreased among second-year residents and were lower than the national average, suggesting that the demanding nature of EM training may contribute to a decline in EI over time. Understanding and addressing this trend is important for maintaining resident emotional resilience and well-being. This aligns with recent calls to formally integrate EI development into professional identity formation curricula throughout medical training [24].

Papanagnou et al. also reported that well-being tends to decline with ongoing care of acutely ill patients, recommending longitudinal studies and targeted interventions to enhance resident well-being [23]. Consistent with these observations​​​​​​, our study found that the well-being sub-score of EI was positively associated with acceptance into EM programs, underscoring the importance of interpreting EI scores comprehensively and emphasizing well-being as a critical component of both selection and training.

A notable strength of this study is its geographic diversity, encompassing applicants from all regions of Saudi Arabia and providing broad national representation. It was also a whole-population study, including all SBEM applicants for the 2019 match rather than a limited sample, enhancing generalizability. The use of well-validated instruments - the NEO-FFI-3 and TEIQue-SF - further strengthens the study’s reliability.

Overall, our findings indicate that academic performance remains the dominant factor influencing acceptance into Saudi EM residency programs, overshadowing non-cognitive traits such as EI and personality. This highlights an opportunity to refine the selection process by incorporating structured, standardized evaluations of EI and personality, ensuring that interpersonal and emotional competencies essential for success in EM are appropriately recognized during residency selection.

Limitations

This study has several limitations. First, the findings are specific to the 2019 recruitment cycle and may not be generalizable to other years, as selection criteria and applicant pools can vary over time. Second, several variables demonstrated p-values between 0.05 and 0.20 (e.g., gender, SMLE score, and extraversion in the univariate analysis of the overall sample; marital status and emotionality in the multivariate analysis; and fitness scores from the Riyadh subgroup interviews), suggesting these variables might reach statistical significance in larger or multiyear cohorts. Third, the interview score data were available only for a single training center, limiting the generalizability of the post hoc subgroup findings. Future research should replicate this study across different recruitment cycles and multiple training centers to confirm the stability of these associations.

## Conclusions

In this study, EI was not independently associated with the likelihood of acceptance into EM residency programs. These findings underscore the continued weight given to cognitive metrics, particularly the CS and GPA, in EM residency selection in Saudi Arabia. However, they should not be read as an argument against assessing non-cognitive attributes. Rather, they highlight a gap between the value PDs place on personality and interview impression and the lack of structured tools currently used to measure these constructs. Greater emphasis should be placed on developing and validating structured methods to evaluate EI and personality among EM residency candidates to ensure a more comprehensive selection process and potentially enhance both program and candidate outcomes.
